# Supplementary material for: Mitochondrial fusion but not fission regulates larval growth and synaptic development through steroid hormone production
Source: eLife. 2014 Oct 14;3:e03558. doi: 10.7554/eLife.03558 (PMC4215535; doi:10.7554/eLife.03558)
Supplement: Figure 2—source data 1. — Lethal staging of Drp1 transheterozygous combinations of Drp1KG38015, Drp1[T26] and Drp1 with Drp12 mutant alleles. DOI: http://dx.doi.org/10.7554/eLife.03558.006 [file elife03558s001.pdf]

Figure 2-source data 1

Lethal stage analysis of *Drp1* mutant alleles				
***Drp1* Alleles**	***Drp1***^{1}	***Drp1***^{2}	***Drp1***^{KG38015}	***Drp1***$^{[T26]}$
Drp1^{1}	L1	Pupae	ND	ND
Drp1^{2}	Pupae	Pupae†	L3	L3
Drp1^{KG38015}	ND	L3	Embryo	L1
Drp1$^{[T26]}$	ND	L3	L1	L1

L3=Third instar larvae, †=Adult Escapers, ND=Not determined and L1=First instar Larvae
