# Supplementary material for: Mitochondrial fusion but not fission regulates larval growth and synaptic development through steroid hormone production
Source: eLife. 2014 Oct 14;3:e03558. doi: 10.7554/eLife.03558 (PMC4215535; doi:10.7554/eLife.03558)
Supplement: Figure 2—source data 2. — Phenotypic comparison table of Marf, Drp1 and Marf and Drp1 mutants in mitochondria morphology, mitochondria membrane potential (MMP), ATP levels, ROS (DHE) intensity, bouton numbers and 20-hydroxyecdysone (20E) levels. Figure 2B MarfB panel has both puncta globular (P) and non-puncta (NP) staining that were both used to measure MMP. MMP, ATP levels and ROS intensity were normalized to controls and all columns are representative of three independent experiments ± SEM. DOI: http://dx.doi.org/10.7554/eLife.03558.007 [file elife03558s002.pdf]

Figure 2-source data 2

Phenotypic analysis of *Marf* and *Drp1* mutant alleles						
**Alleles/ Experimental Assays**	**Mitochondria Morphology**	**MMP**	**ATP Levels**	**ROS Intensity**	**Bouton Quantification**	**20E Levels**
**Control**	-	1±0.05	0.97±0.06	1±0.08	108±7.57	25.5±2.42
$Marf^B$	Fragmented	P=1.1±0.03<br>NP=0.6±0.06	0.57±0.03	2.7±0.23	194±9.06	6.61±0.22
$Drp1^2$	Fused	1.2±0.06	0.63±0.09	1.78±0.14	99±8.28	28.3±1.85
$Drp1^1/Drp1^2$	Fused	1.3±0.11	0.58±0.03	1.85±0.18	112±10.40	22.1±2.27
$Drp1^{[T26]}/Drp1^2$	Fused	1.2±0.01	0.57±0.05	1.94±0.22	104±6.05	24.6±1.92
$Drp1^{KG38015}/Drp1^2$	Fused	1.3±0.11	0.54±0.02	2.1±0.09	103±5.46	23.8±1.97
$Marf^B;Drp1^2$	Fragmented	P=1.1±0.08<br>NP=0.5±0.04	0.41±0.04	4.61±0.24	209±11.43	7.32±0.68

MMP=Mitochondria Membrane Potential, ROS=Reactive Oxygen Species, 20E= 20-hydroxyecdysone P=Punctate, NP=Not Punctate, MMP, ATP levels and ROS intensity were normalized to controls and all columns are representative of three independent experiments with SEM.
