# Supplementary material for: Mitochondrial fusion but not fission regulates larval growth and synaptic development through steroid hormone production
Source: eLife. 2014 Oct 14;3:e03558. doi: 10.7554/eLife.03558 (PMC4215535; doi:10.7554/eLife.03558)
Supplement: Figure 5—source data 1. — Tissue specific Gal4 screen using UAS-Marf to assess rescuing ability of the Marf mutant lethal stage and bouton morphology phenotypes. Ubiquitous expression of Marf resulted in rescue of both lethality and bouton phenotype in Marf mutant, while RG specific expression of Marf rescues the Marf mutant bouton phenotype. DOI: http://dx.doi.org/10.7554/eLife.03558.013 [file elife03558s003.pdf]

Figure 5-source data 1

Tissue Gal4 screen for rescue of *Marf*^{B} mutants lethality and bouton morphology			
**Tissue Expression†**	***Gal4* line(s)**	**Lethality**	**Bouton Morphology**
Ubiquitous	*Actin* or *Tubulin*	Adult	Normal
Neuron	*D42* or *Nsyb*	L3*	Increased and small boutons
Muscle	*C57* or *Mef2*	L3*	Increased and small boutons
Neuron and Muscle	*D42* and *Mef2*	L3*	Increased and small boutons
Glial	*Repo*	L3*	Increased and small boutons
Ring gland (RG)	*Feb36* or *Phm*	Pupa*	Normal

† = Tissue expression based on Bloomington stock report

L3 = Third instar larva stage and * = Lengthen third instar larva stage
