# Supplementary material for: Mitochondrial fusion but not fission regulates larval growth and synaptic development through steroid hormone production
Source: eLife. 2014 Oct 14;3:e03558. doi: 10.7554/eLife.03558 (PMC4215535; doi:10.7554/eLife.03558)
Supplement: Figure 5—source data 2. — Tissue specific Gal4 screen using Marf IR for phenocopying Marf mutant lethal stage and bouton morphology phenotypes. Ubiquitous knockdown of Marf resulted in both prolonged third instar larval stage and similar Marf mutant bouton phenotype, while RG specific knockdown of Marf phenocopied the Marf mutant bouton phenotype. DOI: http://dx.doi.org/10.7554/eLife.03558.014 [file elife03558s004.pdf]

Figure 5-source data 2

Tissue Gal4 screen for lethality and alterations to bouton morphology			
**Tissue Expression**†	***Gal4* line(s)**	**Lethality**	**Bouton Morphology**
Ubiquitous	*Actin* or *Tubulin*	L3*	Increased and small boutons
Neuron	*D42* or *Nsyb*	Pupa	Normal
Muscle	*C57* or *Mef2*	Adult	Normal
Neuron and Muscle	*D42* and *Mef2*	Pupa	Normal
Glial	*Repo*	Adult	Normal
Fat Body	*Lsp2*, *r4* or *Cg*	Adult	Normal
Haemocytes	*Cg*	Adult	Normal
Oenocytes	*Ok376* or *Ok72*	Adult	Normal
Ring gland (RG)	*Feb36*, *Mai60* or *Phm*	Pupa*	Increased and small boutons

† = Tissue expression based on Bloomington stock report

L3 = Third instar larva stage and * = Lengthen third instar larva stage
